# Membrane vectorial lipidomic features of coral host cells’ plasma membrane and lipid profiles of their endosymbionts *Cladocopium*

**DOI:** 10.1038/s42003-024-06578-8

**Published:** 2024-07-18

**Authors:** Tatyana V. Sikorskaya, Ekaterina V. Ermolenko, Taliya T. Ginanova, Andrey V. Boroda, Kseniya V. Efimova, Mikhail Bogdanov

**Affiliations:** 1 A.V. Zhirmunsky National Scientific Center of Marine Biology, Far Eastern Branch, Russian Academy of Sciences, Vladivostok, Russian Federation; 2 Department of Biochemistry and Molecular Biology, the University of Texas Health Science Center, McGovern Medical School, Houston, TX USA

**Keywords:** Lipidomics, Membrane structure and assembly

## Abstract

The symbiotic relationships between coral animal host and autotrophic dinoflagellates are based on the mutual exchange and tight control of nutritional inputs supporting successful growth. The corals *Sinularia heterospiculata* and *Acropora aspera* were cultivated using a flow-through circulation system supplying seawater during cold and warm seasons of the year, then sorted into host cells and symbionts and subjected to phylogenetic, morphological, and advanced lipid analyses. Here we show, that the lipidomes of the dinoflagellates *Cladocopium* C1/C3 and acroporide-specific *Cladocopium* hosted by the corals, are determined by lipidomic features of different thermosensitivity and unique betaine- and phospholipid molecular species. Phosphatidylserines and ceramiaminoethylphosphonates are not detected in the symbionts and predominantly localized on the inner leaflet of the *S. heterospiculata* host plasma membrane. The transmembrane distribution of phosphatidylethanolamines of *S. heterospiculata* host changes during different seasons of the year, possibly contributing to mutualistic nutritional exchange across this membrane complex to provide the host with a secure adaptive mechanism and ecological benefits.

## Abstract (web summary)

An advanced lipidomic study of corals *Sinularia heterospiculata* and *Acropora aspera* showed that plasma membrane of host cells has unique vectorial lipidomic features and compositionally distinct lipid profile from their endosymbiotic dinoflagellates.

## Introduction

Organisms of the phylum Cnidaria, including corals, are primitive aquatic animals known for their unique feature of having stinging cells (cnidocytes)^1^. Many corals establish complex relationships with a wide range of microorganisms that constitute an important part of the coral holobiont. The best-known coral symbionts are intracellular dinoflagellates of the family Symbiodiniaceae. The formation of symbiosomal cells in cnidarians is regarded as phagocytosis where the innate immunity of the coral host is suppressed for an indefinite period. A lysosomal-like organelle inside corals’s gastrodermal cells contains a dinoflagellate cell which provides the coral host with diverse photosynthetic products^2^.

The plasma membrane, where many proteins with various functions are localized, is particularly important for determining cell shape and serves as the first line of defense against adverse environmental factors. The structure and mechanical properties of the plasma membrane are mainly determined by the glycerophospholipid (PL) bilayer^3^. Coral PL include phosphatidylethanolamine (PE), phosphatidylcholine (PC), phosphatidylserine (PS), phosphatidylinositol (PI), and ceramideaminoethylphosphonate (CAEP) with amino-alcohol backbone rather than glycerol structure^4^. Membrane lipids of coral symbionts, in addition to PL, include extra-plastidic betaine lipids (BL) 1,2-diacylglyceryl-*sn*-glycero-3-*O*-carboxy-(hydroxymethyl)-choline (DGCC), anionic phosphatidylglycerol (PG), sulfolipid sulfoquinovosyldiacylglycerols (SQDG), and neutral glycolipids (GL) such as monogalactosyldiacylglycerols and digalactosyldiacylglycerols (MGDG and DGDG, respectively), formed the membrane of the photosynthetic apparatus in Symbiodiniaceae^5^. Due to rapid advances in high-performance liquid chromatography and mass spectrometry (HPLC–MS), detailed information on PL molecular species of marine organisms has become increasingly available^4^. Lipids of symbiotic coral species are a mixture of lipids from both symbionts and their host. Due to the complexity of the organism, membrane lipids of the whole coral-symbiotic organism are studied^6–9^. There are a few studies considering membrane lipid molecular species of isolated Symbiodiniaceae^10,11^; however, studies on membrane lipids of coral host are extremely scarce.

The heterogeneous lipid composition of a biological membrane determines physical properties that modulate cell signaling and protein functions. The specific location of PL in the biomembrane bilayer is reported to be linked to the processes of apoptosis^10^, oncogenesis^12^, and cell division^13^ and function of proteins residing in the membrane^14^. The trans-bilayer distribution of PL can be specific for various organisms. In the plasma membranes of eukaryotes, PE and PS are present in the inner leaflet of the bilayer membrane^15^. The inner leaflet of plasma membrane in *Bacillus subtilis*^16^, as well as the plasma leaflets of inner and the periplasmic leaflet of the outer membrane in Gram-negative bacteria, are enriched in PE^17^. Due to permanent or transient asymmetric distribution of lipid head groups and acyl chain unsaturation, molecular (number of fatty acids, their length and saturation) and compositional asymmetry is closely associated with the physical asymmetry of the bilayer resulting in a less fluid, more tightly packed outer leaflet, and a more fluid, loosely packed inner leaflet in eukaryotic plasma membranes resulting in fluidity gradient across bilayer. Molecular and compositional asymmetries are closely associated with physical asymmetries of bilayers^18^ in both eucaryotic^19^ and procaryotic cells^17^; however, this phenomenon is mirrored in prokaryotic plasma membranes^17^. Moreover, two general mechanisms (flippase-free or flippase-guided) for controlling the asymmetric distribution of lipids across the membrane can coexist in nature^20^. Although biological membranes are most commonly asymmetric, the asymmetry is, however, not absolute^19^ and symmetric biological membranes appear to exist in the nature. For instance, PL of *Drosophila* cells are equally distributed between the inner and outer leaflets of plasma membrane. The constitutive scrambling of insect plasma membrane lipids results in symmetric lipid distribution and an extremally deformable membrane^21^. Therefore, new explanatory hypotheses and experimental models should be explored to understand how widely the transmembrane heterogeneity is distributed among different forms of life. Systematic empirical research and development of different vectorial probes (vectorial lipidomics) should revolutionize our understanding of the physiological significance of lipid (a)symmetry as one of main architectural principles of membrane assembly and function.

The trans-bilayer distribution of phospholipids in the biomembrane of cnidarian cells has never been reported due to the complex coral system and to a lack of proper molecular tools. Information about the vectorial lipidome is very important as it may help predict the mechanical properties of membranes and contribute to understanding of bilayer-leaflet specific regulations of membrane proteins functions and various cellular processes in cnidarian host and symbionts. Unfortunately, all current studies on cell membrane of cnidarians neither employ vectorial lipidomics nor address the importance of transmembrane distribution of individual lipids.

In order to meet all these demands, the heterogeneous cells of the symbiotic soft coral *Sinularia heterospiculata* (Cnidaria: Anthozoa: Octocorallia: Alcyonacea: Alcyoniidae) and the reef-building coral *Acropora aspera* (Cnidaria: Anthozoa: Hexacorallia: Scleractinia: Acroporidae) were sorted into host cells and poor symbionts and examined by light and transmission electron microscopy (TEM). Membrane lipids of the separated coral cells were analyzed by HPLC–MS. The transmembrane distribution of important PL classes was characterized by vectorial molecular probes. We used 2,4,6-trinitrobenzenesulfonic acid (TNBS), which does not readily penetrate biological or liposomal membranes, and 1,5-difluoro-2,4-dinitobenzene (DFDNB), which labels the free amine groups of lipids in both membrane leaflets due to its high permeability. We chose an approach previously proposed to sequentially apply the TNBS and DFDNB probes^17^ in order to determine head amino, polar and acyl group asymmetries in single membrane systems using mass-spectrometric methods. This made it possible to assess the trans-bilayer distribution of primary amine containing PE, PS, and CAEP in membranes of coral host cells.

## Results

### Phylogenetic and morphological characteristics of host and symbiont cells of *Acropora aspera* and *Sinularia heterospiculata*

#### Acropora aspera

**Fig. 1:**
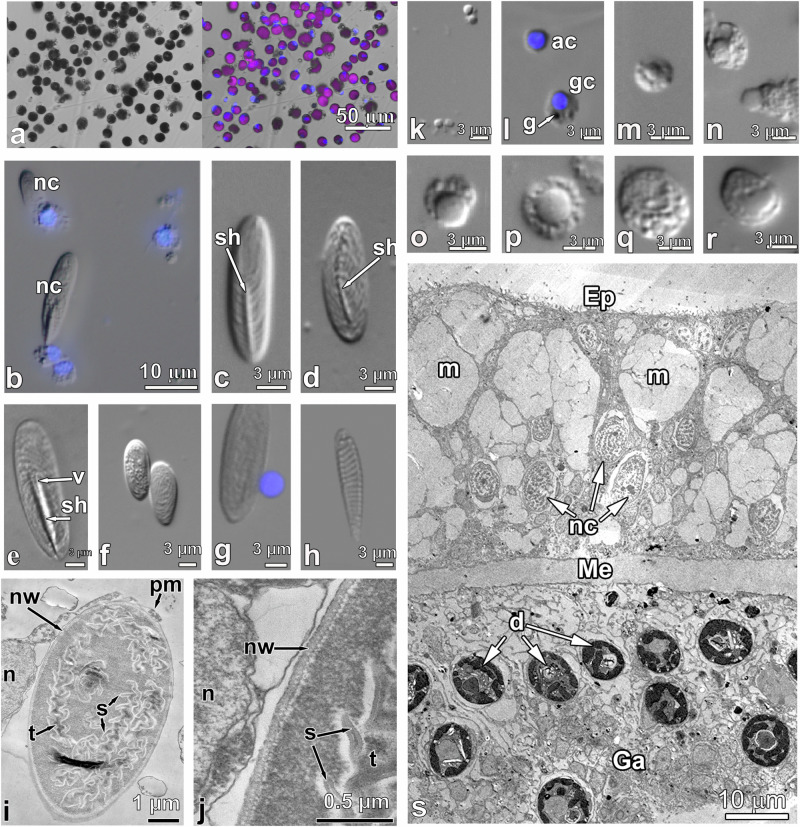
Host cell types and symbionts of *Acropora aspera.* **a** Sorted cell suspension of symbiotic coccoid-shaped dinoflagellates from *A. aspera* (bright field, pink colored autofluorescence in Gy5 channel marks chlorophyll). **b** Nematocysts in host cell suspension. **c**, **d** b-Mastigophores. **e** p-Mastigophore. **f**, **g** Holotrichous isorhizas. **h** Spirocyst. **i**, **j** Transmission electron micrographs of cross sections through nematocyte. **k** Small agranular host cells. **l** Agranular and granular host cells. **m**–**r** Granular host cells. **s** Transmission electron micrographs of cross section through *A. aspera* tentacle. The letter designations are as follows: ac agranular cell, g granule, gc granular cell, n nucleus, nc nematocyst, nw nematocyst’s wall, pm plasma membrane, s spines, sh shaft, t tubule, v V-shaped notch at the end of shaft, Ep epidermis, Ga gastrodermis, Me mesoglea, m mucous cell, nc nematocyst, and d symbiotic dinoflagellates. Blue fluorescence indicates nuclear DNA stained with DAPI.

To study the lipidomic characteristics of certain cell fractions, we sampled the adult symbiotic hexacoral colonies (Fig. 1). Our molecular phylogenetic analyses showed that the *Acropora* specimens shared 99–99.9% sequence similarity with the *A. aspera* sequences from NCBI. For more detailed information, see the Supplementary Data 1 and Supplementary Data 2. After mechanical dissociation, we sorted Symbiodiniaceae cells (Fig. 1a) and host cells (Fig. 1b–r). By mechanical knocking them out with a high-pressure jet, we removed the coral symbionts without host membranes. The suspensions of pure Symbiodiniaceae cells from *A. aspera* contained symbiotic coccoid-shaped dinoflagellates of 9.5–13.5 µm in diameter (Fig. 1a). All colonies of *A. aspera* were characterized by the presence of one Symbiodiniaceae species, the acroporide-specific *Cladocopium*. The Integer NJ Net network, constructed based on the LSU rRNA dataset from *Cladocopium* sequences from this study and from GenBank, is presented in Supplementary Fig. 1 (Supplementary Data 1).

We examined the morphology of the sorted host cells by light microscopy and TEM. Among the host cells of *A. aspera*, nematocytes and spirocytes, also known as stinging cells, were abundant and easily recognizable^22^. These cells contained stinging capsules: nematocysts and spirocysts, respectively (Fig. 1b–h). According to the classification proposed by a number of authors^23,24^, *A. aspera* nematocysts are represented by mastigophores of two varieties (b-mastigophores and p-mastigophores) and holothrichous isorhizas. In our study, mastigophores had a prominent shaft which was a dilated part of the tubule. In p-mastigophore, the base of the shaft had a V-shaped notch, which was absent from b-mastigophore (Fig. 1c–e). The length of b-mastigophore was 15–17 µm, and the width was ca. 4 µm. P-mastigophores were larger, 20–21 µm long and 5–6 µm wide. Isorhizas lacked the shaft; the length and width of their capsules varied from 12 to 17 µm and from 5 to 7 µm, respectively (Fig. 1f, g). Nematocytes contained a thick-walled stinging capsule surrounded by a small layer of cytoplasm (Fig. 1i, j). Cell nuclei were oval or rounded in shape, 2.2–2.5 µm in diameter. The tubule had rows of spines. The tips of the spines were located at equal distances from each other (Fig. 1j). Spirocytes had a thin-walled capsule 8–14 µm long and 2.2–2.5 µm wide. A spirally twisted tubule without spines was visible in the capsule (Fig. 1h). The suspension of *A. aspera* host cells contained very small (up to 3 µm) and small (4–6 µm) agranular cells with dense rounded nuclei of 1.7–1.9 and 2.5–3.5 µm in diameter, respectively (Fig. 1k, l). The cells had a small amount of cytoplasm that did not contain granules. Cytoplasm of a large number of the cells had a granular structure due to the presence of granules, vacuoles, and other inclusions (Fig. 1l–r). Granular cells could be small (3.0–4.5 µm), with nuclei of 1.5–1.9 µm in diameter; cells of medium size 5.0–6.5 µm had nuclei of 2.5–3.5 µm in diameter; large cells 6.5–11.5 µm had nuclei of 2.6–4.5 µm. Granular inclusions could occupy the entire volume of the cell, including the supranuclear cytoplasm (Fig. 1q). In host cell suspensions of the studied corals, different cell types dominated. A significant portion of *S. heterospiculata* host cells apparently consisted of granular amoebocytes. These were actively migrating cells, often found in all tissues of the native coral (Fig. 1o)^1^. Granular amoebocytes were not firmly connected via their contacts with mesoglea fibers and cells and, as a result, were apparently easily released from the coral during mechanical removal and retained their integrity.

#### *Sinularia heterospiculata*

**Fig. 2:**
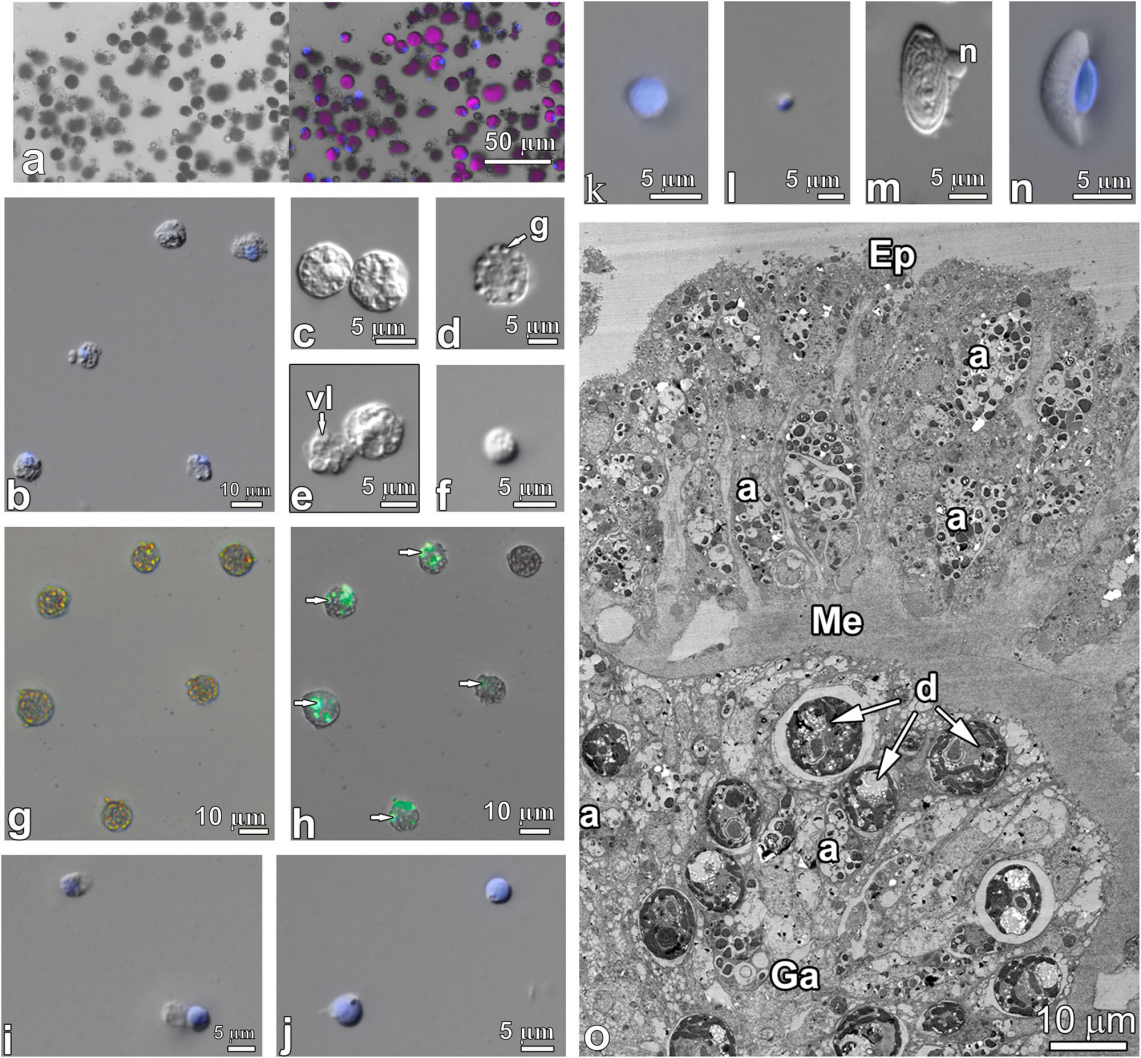
Types of host and symbiont cells from *Sinularia heterospiculata.* **a** Sorted cell suspension of symbiotic coccoid-shaped dinoflagellates from *S. heterospiculata* (bright field, pink colored autofluorescence in Gy5 channel marks chlorophyll). **b**–**f** Granular host cells. **g**, **h** Autofluorescence of some granules in granular host cells; arrows indicate autofluorescent granules (bright field, green autofluorescence in GFP channel marks granules). **i**–**l** Agranular host cells. **m**, **n** Nematocytes. **o** Transmission electron micrographs of cross section through a *S. heterospiculata* tentacle. The letter designations are as follows: g – granule; n – nucleus; vl – vacuole; a – granular amoebocyte; Ep – epidermis; Ga – gastrodermis; Me – mesoglea; and d – symbiotic dinoflagellates. Blue fluorescence indicates nuclear DNA stained with DAPI.

In this study, we also considered the lipidomic characteristics of individual cell fractions from adult colonies of symbiotic octocoral. In our previous study, we identified the octocoral specimens as *S. heterospiculata* and showed that the coral hosted the dinoflagellates *Cladocopium* sp. (subclades C1 and C3)^11^. After mechanical dissociation, we sorted out Symbiodiniaceae cells (Fig. 2a) and host cells (Fig. 2b–n). The suspensions of sorted symbionts cell from *S. heterospiculata* contained symbiotic coccoid-shaped dinoflagellates of 8.5–15.5 µm in diameter. The suspension of *S. heterospiculata* host cells contained many rounded granular cells ranging in size from 6.5 to 14.5 µm (Fig. 2b–e). They usually had rounded nuclei with a diameter from 2.5 to 6.5 µm. The cytoplasm of the cells had a granular structure due to the presence of granules, vacuoles, and other inclusions. The size of the granules in some types of granular cells reached 1.4–1.6 µm (Fig. 2d). The cytoplasm of some cells was filled with large, up to 2 µm, vacuoles (Fig. 2e). Smaller granular cells (5–6 µm) had nuclei from 1.7 to 3.0 µm in diameter and their cytoplasm usually contained single small granules (Fig. 2f). Some inclusions of granular cells were autofluorescent in DAPI (357/447 nm) and GFP (470/525 nm) channels (Fig. 2g, h). The cell suspension in the samples also contained agranular cells, including small (5.0–6.5 µm) and very small (up to 4.5 µm) cells with a thin layer of cytoplasm around the nucleus (Fig. 2i–l). Their nuclei were rounded, with a diameter of 3.0–5.0 and 1.6–1.9 µm, respectively. Nematocytes were sometimes found (Fig. 2m, n). Among them, there were holothrichous isorhizas that had an oval shape, 16–21 µm long and 8–10 µm wide (Fig. 2m).

Unlike the octocoral, the tissues of *A. aspera* did not contain freely mobile amoebocytes in abundance. Their host cell suspension contained numerous nematocytes. In coral tissues of these species, large nematocytes and mucous cells, together with tall supporting cells, formed the outer epidermal layer of the coral and also part of epithelium of the pharynx and tentacles (Fig. 2o). Nematocysts had a thick double wall and consisted of a complex capsular polymer characterized by cysteine-rich peptides such as minicollagens, which are poorly soluble in proteases^25^.

### Lipid molecular profiles of host and symbiont cells from *Acropora aspera* and *Sinularia heterospiculata*

**Fig. 3:**
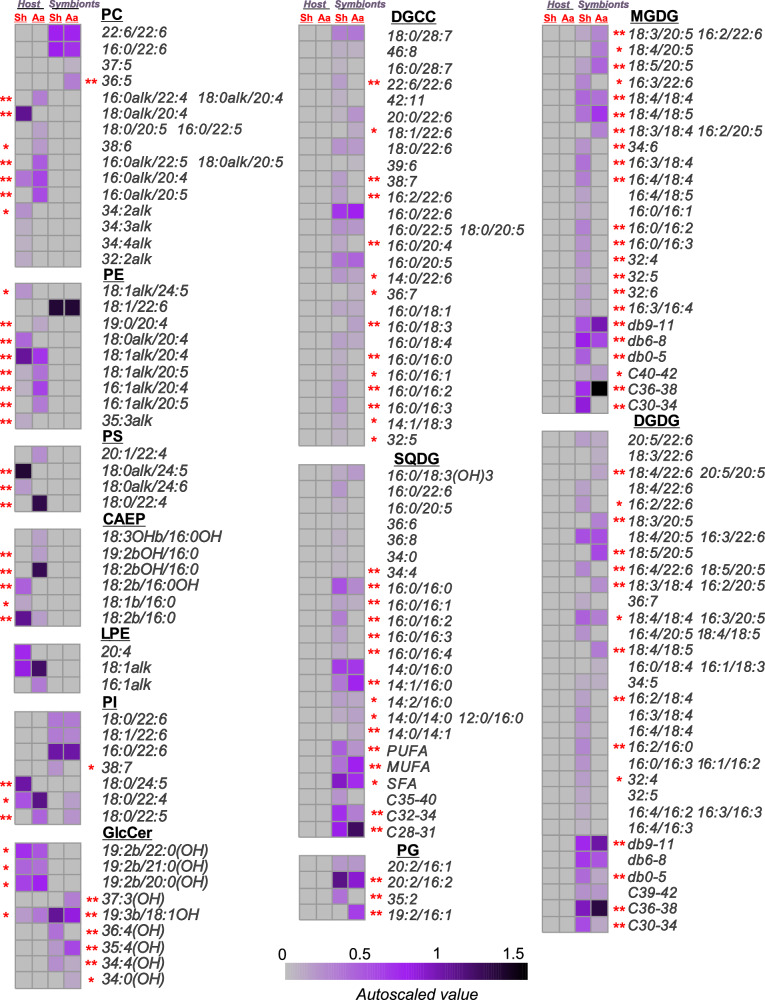
Profiles of lipid molecular species of host and symbionts cells from *Acropora aspera* and *Sinularia heterospiculata.* A heat map of lipid molecular species. The scale bar under the heatmap represents the arcsine-transformed relative abundance of lipid content in the samples. Content values (mean ± SD, *n* = 3 biologically independent samples) are presented as % of lipid class. The significance levels are *p < 0.05 and **p < 0.01 (HSD test) between two coral species separately for host and symbionts. The acronyms are as follows: diacylglycerylcarboxyhydroxymethylcholines (DGCC); sulfoquinovosyl-, monogalactosyl-, and digalactosyldiacylglycerol (SQDG, MGDG, and DGDG); phosphatidylglycerol (PG), phosphatidylethanolamine, phosphatidylcholine, phosphatidylserine, phosphatidylinositol (PE, PC, PS, PI, respectively); lysoPE (LPE); glucosylceramide (GlcCer); and ceramideaminoethylphosphonate (CAEP). Glycerophospholipids that have an alkyl/alkenyl chain attached to the *sn*-1 position by an ether bond are indicated as “alk”.

Using HPLC-MS, we characterized the molecular profiles of membrane lipids separately from coral host and symbiont cell fractions isolated from *A. aspera* and *S. heterospiculata*. (Fig. 3, Supplementary Data 3). GL, PG, and DGCC were surprisingly detected only in the symbionts from the corals *A. aspera* and *S. heterospiculata*. Among GL, a total of 27 and 37 molecular species of MGDG, DGDG, and SQDG were identified in symbionts from *A. aspera* and *S. heterospiculata*, respectively (Fig. 3, Supplementary Data 3). Molecular species MGDG(18:4/18:4), MGDG(18:4/18:5), DGDG(18:4/20:5), DGDG(16:3/22:6), DGDG(18:5/20:5), SQDG(14:0/16:0), SQDG(14:1/16:0), and SQDG(16:0/16:0) were the most abundant GL in the symbiont fractions from both corals. Compared to *A. aspera* symbionts, *S. heterospiculata* symbionts were characterized by a higher content of SQDG with PUFAs (21.75 ± 3.82 in *S. heterospiculata* and 4.43 ± 1.72% of SQDG sum in *A. aspera*; HSD test, *p* = 0.002229) and lower content MGDG (25.78 ± 4.50 in *S. heterospiculata* and 69.46 ± 3.36% of MGDG sum in *A. aspera*; HSD test, *p* = 0.000438) and DGDG (*S. heterospiculata –* 45.49 ± 5.28 and *A. aspera –* 74.34 ± 4.54% of DGDG sum, HSD test, *p* = 0.002214) molecular species with higher unsaturated FAs (9*–*11 double bounds) (Fig. 3, Supplementary Data 3). The most abundant PG molecular species of the symbionts from both coral species consisted of 16:2/20:2. One of the main PG was found to be esterified with one odd-numbered FA, 16:1/19:2 in *A. aspera* symbionts and 35:2 in *S. heterospiculata* symbionts (Fig. 3, Supplementary Data 3). The most abundant DGCC molecular species were 16:0/22:6, 16:0/20:5, and 18:0/28:7 in *A. aspera* and *S. heterospiculata* symbionts.

Such lipids as PC, PI, and glucosylceramides (GlcCer) were present in both host and symbiont cell fractions from the corals *A. aspera* and *S. heterospiculata* (Fig. 3, Supplementary Data 3). In *A. aspera* host cells, ether (an alkyl/alkenyl chain attached to the *sn*-1 position by an ether bond as described for mammalian systems^26^) and diacyl molecular species of PC with C_20_ and C_22_ PUFAs were detected. Only ether molecular species of PC with 20:4 PUFA were detected in *S. heterospiculata* host cells (Fig. 3, Supplementary Data 2). The most abundant PI molecular species were those in the diacyl form with 24:5 PUFA in *S. heterospiculata* host cells and with C_22_ PUFAs in *A. aspera* host cells. In the symbionts from both corals, only diacyl molecular species of PC and PI with 22:6 PUFA were detected. GlcCer molecular species of host were different from that of symbionts in chain length. The most abundant GlcCer of the studied host cell fractions were 19:3b/18:1(OH) and 35:4(OH) for *A. aspera* host and 36:4(OH) for *S. heterospiculata* host. The most abundant GlcCer of the symbionts from both studied corals were 19:2b/20:0(OH) and 19:2b/22:0(OH). Previously, a mixture of 2-(2-hydroxydodecasanoylamino)-, 2-(2-hydroxyuncosanoylamino)-, and 2-(2-hydroxyeicosanoylamino)-1-(β-D-glucopyranosyloxy)-*erythro*-9-methyloctadeca-4*E*-8*Z*-dien-3-ols had been found in the octocoral *Cladiella* sp^27^.

Interestingly and unexpectedly, aminolipids PS, LPE, and CAEP were detected only in coral host lipids (Fig. 3, Supplementary Data 3). Only PE 18:1/22:6 were detected in symbiont lipids. In the host cell fractions of both corals, more than 98% of PE molecular species were in the ether form and contained C_20_ PUFAs. One of the PE molecular species was found with one odd-numbered acyl/alkyl chain, 19:0/20:4 in the *A. aspera* host and 35:3alk in the *S. heterospiculata* host (Fig. 3, Supplementary Data 3). It is worth nothing that the PS molecular species in the *A. aspera* host cell fraction was only in the diacyl form with 22:4 PUFA, and in the *S. heterospiculata* host cell fraction it was in the ether form with C_24_ PUFAs CAEP with hydroxylated sphingosine base was detected in *A. aspera* and CAEP with hydroxylated FAs – in the *S. heterospiculata* host. The most abundant CAEP molecular species were 18:2b(OH)/18:0 in *A. aspera* and 18:2b/16:0 and 18:2b/16:0(OH) – in the *S. heterospiculata* host.

### Transmembrane distribution of aminolipid molecular species of *Sinularia heterospiculata*

We were next interested in examining the transmembrane distribution of lipids in the corals. However, corals produce large amounts of mucus secretions^28^. In *A. aspera*, the presence of a significant number of nematocytes, as well as a large amount of mucus secreted by mucous cells during extraction (Fig. 2o), resulted in difficulties with getting lipids accessible/available for covalent modification. Thus, TNP and DNP-derivatives of lipids in cells of the *A. aspera* host were not detected in a sufficient amount for full identification. In the coral symbionts, PS, and CAEP were not detected and PE(18:1/22:6) was the only molecular species of PE, and, therefore, the aminolipid labeling was applicable only to the coral host cells of *S. heterospiculata*.

For the assessment of aminolipid transmembrane distribution, we sequentially applied the probes TNBS (water soluble and membrane non-permeant) and DFDNB (DMSO soluble and permeant) with different membrane-penetrating properties in order to selectively label aminolipids (PE, PS, and CAEP) within the outer and inner leaflets of coral host cells, respectively. This approach was originally designed and developed for the investigation of dynamics and steady-state distribution of aminolipids in any cell or cellular organelle surrounded by a single membrane^17^. Theoretically DFDNB can also derivatize aminolipids located in the intracellular organelles unless it is used up by dinitrophenylation of plasma membrane aminolipids and further diluted in the internal volume of the cytoplasm. In this case, aminolipids of intracellular membranes will not be labeled due to low intracellular concentration especially in cytoplasm and to the intracellular pH which is maintained close to neutrality (7.0) as a vital parameter, while modification of primary amines by DFDNB (dinitrophenylation) is favored at slightly alkaline or alkaline pH (7.5 and higher). Nevertheless, to avoid this potential problem, cytoplasmically exposed DFDNB was scavenged by methylamine (CH_3_NH_2_) which instantly penetrates plasma membrane^29^. Since no difference in the ratio of trinitrophenol (TNP) and dinitrophenyl (DNP)-derivatives of amino lipids before or after such pretreatment was found in all experiments presented below, we expect that cytoplasmic DFDNB was either fully scavenged by methylamine and /or its efficient concentration was very low.

**Fig. 4:**
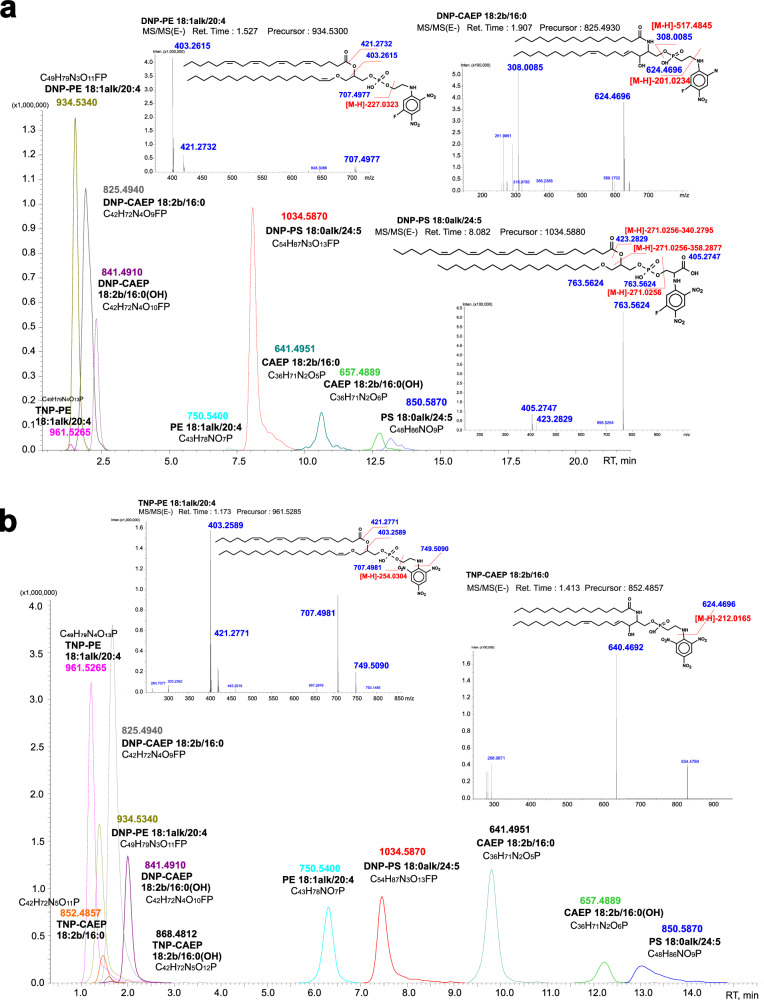
The HPLC–MS analyses of modified lipids of the *S. heterospiculata* host cells. Chromatography–mass spectrometry analysis of DNP- and TNP-derivatives of lipid molecular species of the host soft coral *Sinularia heterospiculata* in the warm (**a**) and cold (**b**) seasons of the year: chromatogram (HPLC-MS (IT-TOF)) of lipid molecular species; mass spectra MS/MS(E–) and fragmentation schemes of lipid DNP- and TNP-derivatives. The acronyms are as follows: phosphatidylethanolamine and phosphatidylserine, (PE and PS); ceramideaminoethylphosphonate (CAEP); DNP- and TNP- derivatives are 1,5-difluoro-2,4-dinitobenzene (DFDNB) and 2,4,6-trinitrobenzenesulfonic acid (TNBS), respectively. The position of C_8_ double bond in the sphingosine base of CAEP is indicated conventionally.

The HPLC–MS analyses of modified lipids of the *S. heterospiculata* host are presented in Fig. 4. TNP-PE and DNP-PE were detected using normal phase HPLC–MS in negative mode. The DNP-PE molecular species with RT 1.527 min gave an ion [M-H]^−^ at m/z 934.5340 that corresponded to the composition [C_49_H_79_N_3_O_11_FP-H]^−^ (calculated 934.5363) (Fig. 4a). The MS^2^ fragmentation of this ion (Fig. 4a) resulted in the formation of ions at m/z 707.4977 and m/z 403.2615 that corresponded to elimination of C_8_H_6_N_3_O_4_F and C_8_H_6_N_3_O_4_F with the 20:4 acyl fragment, respectively. Based on the calculated elemental composition and the value of monoisotopic molecular weight, this molecular species was identified as DNP-PE(18:1alk/20:4). The DNP-CAEP molecular species with RT 1.907 min gave an ion [M-H]^−^ at m/z 825.4940 that corresponded to the composition [C_42_H_72_N_4_O_9_FP-H]^−^ (calculated 825.4948) (Fig. 4a). The MS^2^ fragmentation of this ion (Fig. 4a) resulted in the formation of ions at m/z 624.4696 and m/z 308.0085 that corresponded to elimination of C_6_H_4_N_3_O_4_F and sphingolipid fragment C_34_H_63_NO_2_, respectively. We identified these molecular species as DNP-CAEP(18:2b/16:0). DNP-CAEP(18:2b/16:0(OH)) with RT 2.318 min showed a similar fragmentation (Fig. 4a). DNP-PS molecular species with RT 8.082 min gave a negative ion [M-H]^−^ at m/z 1034.5870 that corresponded to the composition [C_54_H_87_N_3_O_13_FP-H]^−^ (calculated 1034.5888) (Fig. 4a). The MS^2^ fragmentation of this ion (Fig. 4a) resulted in the formation of ions at m/z 763.5624, m/z 405.2747, and m/z 423.2829 that corresponded to elimination of C_9_H_6_N_3_O_6_F, C_9_H_6_N_3_O_6_F with ketene, and C_9_H_6_N_3_O_6_F with 24:5 acyl fragment, respectively. We identified this molecular species as DNP-PS(18:0alk/24:5). DNP-PS(18:0alk/24:6) with RT 8.080 min showed a similar fragmentation.

The TNP-PE molecular species with RT 2.2 min gave an ion [M-H]^−^ at m/z 961.5265 that corresponding to the composition [C_49_H_79_N_4_O_13_P-H]^−^ (calculated 961.5308) (Fig. 4b). The MS^2^ fragmentation of this ion (Fig. 4b) resulted in the formation of ions at m/z 707.4981, m/z 749.5090, m/z 403.2589 and m/z 421.2771 that corresponded to elimination of C_8_H_7_N_4_O_6_, C_6_H_2_N_3_O_6_, C_8_H_7_N_4_O_6_ with the 20:4 acyl fragment and C_6_H_2_N_3_O_6_ with the ketene, respectively. Based on the calculated elemental composition and the value of monoisotopic molecular weight, this molecular species were identified as TNP-PE(18:1alk/20:4). The TNP-CAEP molecular species with RT 1.933 min gave an ion [M-H]^−^ at m/z 852.4857 that corresponded to the composition [C_42_H_72_N_5_O_11_P-H]^−^ (calculated 852.4893) (Fig. 4b). The MS^2^ fragmentation of this ion (Fig. 4b) resulted in the formation of ions at m/z 640.4692 that corresponded to elimination of C_6_H_2_N_3_O_6_. We identified these molecular species as TNP-CAEP(18:2b/16:0). In addition, unreacted PE(18:1alk/20:4), PS(18:0alk/24:5), CAEP(18:2b/16:0), and CAEP(18:2b/16:0(OH)) were also detected (Fig. 4a, b).

The sampling of corals for the experiment on labeling aminolipids in the plasma membrane of coral host cells was carried out in different seasons of the year. The symbiotic soft coral *S. heterospiculata* and the reef-building coral *A. aspera* were cultivated using flow-through circulation system which supplied seawater during warm (May – October) and cold seasons of the year (November–April). Percentages of TNP-, DNP- derivatives and non-derivatized PL molecular species were calculated on the basis of the peak area of negative ions [M–H]^–^ (Supplementary Data 3). In August, TNP-derivatives of PE were detected only for in small amounts 1.21% of sum TNP-, DNP- and non-derivatized PE (Fig. 4a, Supplementary Data 3). In March, the content of TNP-PE was 49.42% of total TNP-, DNP- and non-derivatized PE (Fig. 4b, Supplementary Data 3) demonstrating that this aminolipid is surface exposed in host cells. The reaction with the probes TNBS and DFDNB was the worst for CAEP molecular species, and relative contents of their derivatives varied slightly depending on the sampling season (Fig. 4a, b, Supplementary Data 3). In contrast to PE and CAEP, TNP-PS were not detected in any of the samples (Fig. 4a, b, Supplementary Data 3), which independently confirmed that the integrity of host cell plasma membrane was not compromised under the indicated experimental conditions in such a way that the reaction reached saturation of available primary amines and TNBS could label all the surface-accessible amino groups in the outer leaflet of plasma membrane^17^.

## Discussion

### Lipidome features of symbiotic dinoflagellates

In this study, we managed to clearly verify the lipid compositions of individual members of the coral holobiont. Algae membrane lipids mainly contain glycerolipids: PL (PC, PE, PI and PG), GL synthesized in chloroplasts, and the extra-plastid lipid class BL^5^. Ether lipids (an alkyl/alkenyl chain attached to the *sn*-1 position by an ether bond) have not been detected in algae to date^26^. Our study shows that membrane lipids of the symbiotic dinoflagellates *Cladocopium* C1/C3 and the acroporide-specific *Cladocopium* from the studied corals are composed of diacyl molecular species of PL and GL. We did not find lipids with a primary amino group (PS and CAEP) among the lipids of the symbiotic dinoflagellates and, therefore, these classes of lipids are markers for the host in the symbiotic coral. The major molecular species of PL are esterified with 20:5*n*-3 and 22:6*n*-3 PUFAs. High levels are observed in lipids of Symbiodiniaceae cells isolated from corals^30,31^. The most important *n*-3 PUFAs (20:5*n*-3 and 22:6*n*-3) are not present in higher plants but occur at high concentrations in algae that may be a primary source of these PUFAs in fish^32^. A feature that distinguishes PUFAs from less unsaturated FAs is the presence of a repeating double bond unit that produces an extremely flexible structure rapidly isomerizing to approach the transition state conformation. As a PL component, it has a strong influence on the properties of all membranes, modulating their structure and function^33^. DGCC with extra-long-chain 28:7 PUFA was detected in the coral symbionts. Earlier, C_28_ were identified in Baltic herring and in marine dinoflagellate^34^, including symbiotic dinoflagellates from the corals *S. flexibilis*, *Acropora* sp., and *M. platyphylla*^35^. The lack of any obvious precursors makes it difficult to elucidate how these compounds can be synthesized^34^. Probably, these DGCC are a separate DGCC pool that performs a special function in symbiotic dinoflagellates^35^.

The functional and significant differences in thylakoid lipidome among within the same genus of Symbiodiniaceae are likely associated with the light affinity and temperature tolerance^36–38^. The cells regulate their lipid composition to achieve constant membrane fluidity under various temperature. Normally the polar head group composition did not change significantly, while the acyl chain composition of the membrane lipids varied with the growth temperature; the fraction of unsaturated chains decreased, and the fraction of saturated chains increased, when the growth temperature was increased. As a consequence of the change of the acyl chain structures, the temperature for the lamellar gel to liquid crystalline phase transition is changed simultaneously, and organism to be able to grow in a “window” between a lamellar gel phase and reversed non-lamellar phases (HII). Elevated temperatures can disrupt the normal physiological functioning of photosynthetic organisms by altering the fluidity and permeability of chloroplast membranes that is defined and regulated by their lipid composition. Since SQDG mainly contains saturated acyl chains, while galactolipids (MGDG and DGDG) are highly unsaturated, these classes of thylakoid membrane lipids of symbiotic dinoflagellates need to be considered by analyzing native molecular species rather than total FAs^38^. The symbionts *Cladocopium* C1/C3 from the studied coral *S. heterospiculata* are characterized by a lower degree of galactolipid unsaturation, which, as previously shown, is a characteristic lipidomic feature for these thermosensitive symbionts^10,11,30,38^. SQDG plays especially important role in providing the thermosensitivity of coral symbionts is assigned to, in particular through putative photosystem II interactions^10^. Earlier, we showed that the thermosensitive *Cladocopium* C3 is characterized by a higher content of SQDG with PUFAs^38^, similarly to the lipidome features of the symbionts *Cladocopium* C1/C3 from the studied coral *S. heterospiculata*. Our study has shown that the thylakoid lipid composition differed between distinct *Cladocopium* genotypes. In contrast to *Cladocopium* C1/C3, acroporide-specific *Cladocopium* from the coral *A. aspera* showed lipidome features such as the presence of SQDG with shorter FAs, SQDG with SFAs and MUFAs, and more unsaturated galactolipids like those in *Cladocopium* C3u and C71/C71a from *Acropora* sp^38^. These lipidomic features are characteristic of the thermotolerant dinoflagellates *Durusdinium trenchii*^10,11^. It has been suggested that the thermosensitivity of coral symbionts depends on the lipidome of their thylakoid membranes, which influences photosynthetic performance in photosynthetic organisms^39^. The different thermosensitivity of *Cladocopium* C1/C3 and acroporide-specific *Cladocopium* will undoubtedly have different effects on the survival of the soft corals *S. heterospiculata* and the reef-building coral *A. aspera* under stressful environmental conditions.

PG is considered a vital lipid mainly for its role as a cofactor of photosystems^40^. The PG molecular species of the studied symbionts include 16:1 and 16:2 FAs at one of the glycerol backbones positions. Earlier, 16:1*n*-7, 16:1*n*-9, 16:2, and 20:2*n*-6 FAs were found in lipids of isolated coral symbionts^41,42^. At another position of the glycerol backbone, 19:2 and 20:2 PUFAs were detected in PG of the studied coral symbionts. Botana et al. ^30^ observed the same PG molecular species, 16:1/19:2 and 16:2/20:2, in cultivated symbiotic dinoflagellates. The unusual FA composition of PG may indicate its special properties for coral symbionts. Rather than play structure-forming role for coral symbionts, unusual PG molecules can contribute to stability and photochemical activities of light-harvesting complexes^43^.

### Lipidome features of coral host cells

The *S. heterospiculata* host cells are characterized by a higher content of ether form of PE. The same lipidomic feature was reported for *Sinularia*, *Capnella*, and *Xenia* species^1,8,44,45^. The *A. aspera* host cells are characterized by a higher content of ether form of PE and PC with C_20_ and C_22_ PUFAs, as well as *A. cerealis* and the zoantharian *Palythoa tuberculosa*^46,47^. Earlier, it was shown that the distribution of ether and diacyl forms of PS molecular species depends on the presence of exoskeleton^37^. In corals with a solid exoskeleton (*Acropora*, *Millepora*, and *Allopora*), only the diacyl forms of PS were identified^6,46^. The same lipidome feature was observed in the studied *A. aspera*. In contrast, *S. heterospiculata* contained only the ether form of PS molecular species. It was previously hypothesized that the matching of alkyl to longer acyl chains in PS molecules is a certain compensatory mechanism responsible for the physical properties of the membrane in soft coral cells^37^. The ether-linked alkyl chains in PL allow tighter packing of the latter in the membrane, which leads to decreased membrane fluidity and increased rigidity^48^.

### Lipid transmembrane distribution of coral host cells

In eukaryotic cells, lipid asymmetry is maintained by the interplay of PL transporters: flippases, floppases and scramblases. The former use energy derived from ATP hydrolysis to flip (out-to-in) or flop (in-to-out) lipids across the bilayer, respectively, whereas scramblases facilitate the bidirectional movement of lipids in an ATP-independent manner^49,50^. The strong plasma membrane asymmetry with respect to PS that exists in living cells is lost during apoptosis when the regulated activation of scramblases rapidly exposes PS on the extracellular leaflet. Cell surface externalization of PS is also a general feature of the phagocytosis of apoptotic cells by macrophages^49^. As the example of *S. heterospiculata* clearly demonstrated, aminolipids PS were not derivatized by TNBS, and accordingly, were located only on the inner (cytoplasmic) leaflet of the plasma membrane. The same transmembrane distribution of PS is observed in human cells and other eucaryotic cells^15,19,43^. Thus, in corals, as in other organisms, the PS is located on the inner leaflet of the membrane bilayer, and this PS asymmetric position is apparently clearly controlled by PL transporters, since otherwise the exposure of PS to the extracellular leaflet will trigger the apoptosis.

The sphingophosphonolipid CAEP is a typical structural lipid class of marine invertebrates^51^ and one of the major membrane lipid classes of cnidarians^37^. The function of sphingophosphonolipids is poorly known. We have shown that CAEP within the outer leaflet are barely detected, whereas CAEP produce DNP-derivatives located on the inner leaflet of the plasma membrane of *S. heterospiculata* host cells. Thus, the location of CAEP on the inner leaflet of the membrane may indicate its protective function, since the presence of a C–P bond in the aminoethylphosphonate of CAEP is known to determine the resistance of CAEP to hydrolytic enzymes^52^.

Singer and Nicolson (1972) proposed a fluid mosaic model of membranes^53^. However, with accumulation of new data, a more complex concept of dynamic ordered micro- and nanodomains (membrane rafts) in biomembranes has been developed^54^. The membrane raft hypothesis has formalized a physicochemical principle for a subtype of such lateral membrane heterogeneity, where the preferential associations between sterol and saturated FA acyl chains of sphingolipids drive the formation of relatively packed (liquid ordered phase (Lo)) membrane domains. On the molecular level PL with highly unsaturated FA acyl chains form non-raft (liquid disordered phase (Ld)) domains^54,55^. We have shown that PL and sphingolipids of host cells of *S. heterospiculata* and *A. aspera* differ in the degree of FA acyl chain unsaturation. These differences may suggest that saturated sphingolipids CAEP and GlcCer of corals are involved in the formation of ordered raft domains, while higher unsaturated PL of corals are involved in the formation of disordered domains.

PE is the second most abundant PL in corals^8^. According to numerous studies, PE, similar to PS, and PI are located in the inner leaflet of plasma membranes of eucaryotic cells^15,19,43^. As the example of *S. heterospiculata* clearly demonstrated, transmembrane distribution of aminolipids PE can be changed and this lipid could externalize. The cause of this phenomenon needs to be clarified, but we, however, hypothesize that observed difference can be due to switching one feeding mode to another, e.g., due to changing in nutritional mode from autotrophic (photosynthesis by symbiotic dinoflagellates) to heterotrophic (predation on phyto- and zooplankton). It is known that symbiotic corals partially compensate for the lack of energy through predation and the rate of heterotrophic energy acquisition in corals may depend on the species, health, food, and habitat conditions^56–59^. In the warm season of the year, it has been shown that PE within the outer leaflet were barely detected, whereas PE produce DNP-derivatives located on the inner leaflet of the membrane of *S. heterospiculata* host cells. During this period of the year, an active increase in the population of phyto- and zooplankton is observed in Peter the Great Bay (Sea of Japan, Russia)^60,61^. In the cold season of the year, we observe the appearance of TNP-derivatives in comparable amounts to DNP-derivatives, that is, PE is surface exposed and located on both bilayer leaflets of the membrane, and therefore most likely symmetrically distributed across plasma membrane making membrane of host cells more deformable^21^ during cold season of year. Why were the changes in transmembrane distribution detected only for PE? Due to its unique geometrical shape and chemical properties of PE can confer membranes with a wide range of bilayer physico-chemical and mechanical responses, including changes in surface charge, elasticity, flexibility, morphology that adapt the cell to any given abiotic (thermal, osmotic or ionic) and biotic stresses. The headgroup size of PE is one of the smallest among PL and in addition to forming a bilayer, PE with unsaturated fatty acyl chains is also capable of forming non-bilayer hexagonal phases^62^, which can explain the involvement of PE in processes such as membrane fusion, membrane budding, exo-, endocytosis, cell division, and autophagy^63^. In the relation to the symbiotic coral, the changes in transmembrane distribution of PE can be involved in processes of inter-partner nutrient exchange via maintenance of proper insertion and transmembrane orientation^64,65^, folding^66^, stability^67^ of membrane proteins, including coral membrane transporters^68^, that may promote the establishment of endosymbiosis or facilitate nutrient exchanges between endosymbiotic dinoflagellates and the host cells. In the absence of PE on desired site of plasma membrane the transporters may not have the correct tertiary structure^66^ or orient correctly^64^ and therefore will not function correctly^69^. The folding, topology and function of host cell transporters can be potentially controlled by changes in asymmetric distribution of PE acting as plasma membrane side specific molecular chaperone^70^ and topological determinant^71^ on extracellular and cytoplasmic sides of membrane respectively.

Coral animal host establish a stable mutualistic endosymbiotic relationship with symbiotic dinoflagellates, where the algae donate their abundant fixed carbon in return for waste inorganic nitrogen from the host^2,72^. Photosynthetically derived nutrients are temporarily stored in lipid droplets and starch granules of symbiotic dinoflagellates, followed by remobilization of carbon into all host tissues, including those distant from the symbionts^73^. The intimate details of the coral–dinoflagellate association that would provide full understanding of the metabolic processes in symbiotic coral are unfortunately out of reach^74^. The plasma membrane, its structure, and properties can undoubtedly take part in the regulation of inter-partner nutrient dynamics and homeostasis. Not only biosynthesis and remodeling of different phospholipids but also their asymmetry is finely regulated, highlighting the importance of these lipids in cell homeostasis and responses during stressful situations. However, research in this area is very limited and the present study is the first attempt to elucidate the transmembrane distribution of lipids in the plasma membrane of the coral host. The observed differences in lipid profiles and transmembrane distribution of amino phospholipids and CAEP of coral endosymbionts and host cells respectively are consistent with architectural and guiding principles contributing to lateral and transmembrane heterogeneity, deformability and rigidity of biological membranes. Our study is the first unambiguous demonstration of aminophospholipid asymmetry and the first to describe a seasonal dynamic change in distribution of PE across the plasma membrane of coral host cells. This phenomenon which will be utilized for future experimental analyses of the role of the transmembrane dynamics of this lipid in coral host in order to unlock further the cellular complexity of coral-dinoflagellate symbiosis and elucidate physiological significance of PE sidedness in coral-symbiont relationships.

There are still few experimental approaches for mapping of lipid asymmetry in living cells^75^. Biochemical technique is a strong approach to study lipid asymmetry. Sequential utilization of two primary amine-specific vectorial non-penetrating (i.e., outer leaflet specific) TNBS and penetrating (DFDNB) probes led to the development of a novel approach for determining head-amino and acyl-group using mass-spectrometric methods (designated by us as vectorial lipidomics)^17^. We developed this approach further and utilized vectorial probes with different intrinsic permeability to assess the trans-bilayer distribution of primary amine containing PE, PS, and CAEP in membranes of multiorganellar coral host cells. We are setting up an experiments on the distribution of other membrane lipids, including PC, PI, sterols, and glycolipids. The corals form one of most biodiverse marine ecosystems. Our study is the first unambiguous demonstration of aminophospholipid asymmetry dynamics in the plasma membrane of coral host cells. We are eager to see our data on the transmembrane distribution of the aminolipids to become available to a wider audience in order to demonstrate feasibility of vectorial lipidomics for the further development of interpartner lipidomic studies of cnidarian-Symbiodiniaceae cells and other multiorganellar organisms.

## Star methods

### Specimen collection and coral culture

Colonies of the octocoral *S. heterospiculata* (Cnidaria: Anthozoa: Octocorallia: Alcyonacea: Alcyoniidae) and the hexacoral *A. aspera* (Cnidaria: Anthozoa: Hexacorallia: Scleractinia: Acroporidae) were cultivated at 27 °C in a 500 L tank. In the experimental tank, a flow-through circulation system was used, which supplied seawater from Peter the Great Bay northwestern Sea of Japan. For illumination, we use white fluorescent lamps (National, FL20SS-N/18) with a photosynthetic photon flux density of 200 µmol photons m^–2^ s^–1^. The photoperiod was 12 h light/12 h dark. The coral colonies were grown to 10 cm in height for 1 year.

### Sequence alignment and molecular phylogenetic analyses

Details of the protocol of DNA extraction from fresh and ethanol-fixed coral colonies are provided by Sikorskaya et al.^11^. For species identification of *Acropora* sp. coral, direct sequencing of the highly polymorphic single-copy nuclear *Pax-C* 46/47 intron (*Pax-C*) was applied. The nuclear *Pax-C* of *Acropora* was amplified with the PaxC_intron-FP1 (5′-TCCAGAGCAGTTAGAGATGCTGG-3′) and PaxC_intron-FR1 (5′- GGCGATTTGAGAACCAAACCTGTA-3′) primers^76^. The presence and specific species of Symbiodiniaceae were verified by PCR screening and sequencing of the obtained products according to the protocols and molecular-genetic markers published by Sikorskaya et al. ^11,38^. The sequence data were deposited in GenBank under the following accession numbers: OR166010-OR166012, OR180049-OR180050. More detailed information is presented in Supplementary Data.

### Isolation and sorting of coral cells

Cells of the *S. heterospiculata* and *A. aspera* hosts and their pure symbionts were mechanically removed from the coral colonies weighing about 2–3 g with a high-pressure jet of 5 mM EDTA in 1.2 µm filtered seawater. About 200 mL of heterogeneous cell suspension, containing pure symbionts (symbiotic dinoflagellates) and various types of host cells, were centrifuged at 700 × *g* for 5 min; the pellet was re-suspended in 10 mL of calcium- and magnesium-free artificial seawater (CMFSW, pH 7.7). The cell suspension was analyzed and separated into fractions on a MA900 cell sorter (Sony Biotechnology, San Jose, CA, USA). Each particle (cell debris, single cell, or cell aggregates) detected by the cell sorter was assumed to be an “event”. Single events made up more than 80% of total events in the suspension and were determined by triangle gating in a FSC-A against FSC-H plot in order to remove cell aggregates and debris from the sorted fraction. Single events with autofluorescence under excitation by light at 488 nm and emission at 695/50BP nm (chlorophyll-positive events) were considered as symbiont cells, while other cells were considered as host cells. Pure symbiotic dinoflagellates and host cells were sorted into separate 15 mL sterile centrifuge tubes using the “Semi-purity” mode of the sorter to reach 5 × 10^6^ symbiont cells and 10 × 10^6^ host cells. The resulting suspensions of sorted cells were centrifuged at 700 × *g* for 5 min, and the pellet was re-suspended in 300 µL of CMFSW. The host and symbiont cell suspensions were examined by light microscopy and TEM. For *S. heterospiculata* and *A. aspera*, symbiont and coral host cell suspensions were taken for lipid extraction (in triplicate). The suspensions of host cells were examined for the transmembrane distribution of aminolipids using the probes TNBS and DFDNB.

### Light microscopy

Live cell suspensions and fixed samples were used to assess the cell composition of the suspensions of separated host cells of *S. heterospiculata* and *A. aspera*. The samples were fixed for 1 h at 4 °C by adding a 25% glutaraldehyde solution to the cell suspensions, so that the final fixative concentration in the sample was 2.5%. Cells were stained with DAPI (GERBU Biotechnic GmbH, Germany) at a final concentration of 1 μg/mL. The live cell suspensions and fixed stained samples were placed in 24-well plates (Nunclon/Thermo Fisher, Illkirch, France), viewed, and photographed using an Evos M5000 imaging system (Thermo Fisher Scientific, USA). Additionally, smears of live and fixed cells were used to examine the host coral cell suspension at a high resolution. To prepare smears, 50 µL of live cell suspension was applied to polylysine-coated slides; the samples were kept for 40 min for cell precipitation. To fix the smears, 25% glutaraldehyde was added to the precipitated cells, so that the final fixative concentration in the sample was 2.5%. Cells were stained with DAPI at a final concentration of 1 µg/mL. The smears with live and fixed cells were analyzed and photographed under an Axio Imager Z2 light fluorescent microscope (Carl Zeiss, Jena) equipped with an AxioCamHRc digital camera (Carl Zeiss).

### Transmission electron microscopy

To study the morphology of native tissues of the corals *S. heterospiculata* and *A. aspera*, about 5 mm^3^ of samples were excised, fixed and examined using a Carl Zeiss Libra 120 transmission electron microscope (TEM) or a Carl Zeiss Sigma 300 Vp scanning electron microscope (SEM) equipped with a STEM-detector as described earlier^1^.

To examine the morphology of sorted host cells, the cell suspension samples were supplemented with 25% glutaraldehyde to a final fixative concentration of 2.5% in the sample. The samples were fixed for 24 h at 4 °C. After the fixation, the material was rinsed in 0.05 M cacodylate buffer at pH 7.5 and embedded in 2% low-melting agarose LE (GERBU) prepared on cacodylate buffer. The samples were post-fixed in 1% OsO_4_ in cacodylate buffer for 1 h, dehydrated in a graded series of ethanol (30, 50, 70, 90, and 96%) and acetone (100%), and embedded in Araldite. The sections were cut with glass knives on an Ultracut UC6 (Leica) ultratome. Ultrathin (60–75 nm) sections were collected on copper grids, stained with uranyl acetate and lead citrate, and examined under a Carl Zeiss Libra 120 TEM or a Carl Zeiss Sigma 300 Vp SEM equipped with a STEM-detector.

### Lipid analysis

A total of 300 µL of suspensions of symbiont and host cells suspension of *S. heterospiculata* and *A. aspera* (four samples) in CMFSW (pH 7.7) were used for lipid extraction. Lipids were extracted by adding three volumes of chloroform: methanol (1:2, v/v) to create a single-phase solution. It was then supplemented by an equal volume of water and left overnight for phase separation at 4 °C. The lower phase was recovered, evaporated, and re-dissolved in 20 µL of chloroform. The membrane non-permeant probe TNBS and the fully permeant amino-specific probe DFDNB were used sequentially to elucidate the aminophospholipid (PE, PS, and CAEP) topography of the coral host cells followed the vectorial lipidomic approach^17^ with some modifications. Prior covalent modification of aminolpids in plasma membrane, the 300-µL aliquots of host cell suspensions of *S. heterospiculata* and *A. aspera* were pretreated with 30 µL of methylamine (CH_3_NH_2_, 0.5 mM) for 5 min which was subsequently washed twice by CMFSW. The aliquots were labeled, first, by 20 µL of 0.1% TNBS (at a final concentration of 0.21 mM) and then by 20 µL of 200 mM DFDNB (at a final concentration of 12.50 mM) at room temperature for 1 h in the dark for both. The aliquots were washed with CMFSW after each labeling step. Then, lipids were extracted as described above. Four non-derivatized and two derivatized lipid extracts were analyzed on a HPLC system with a high-resolution tandem mass spectrometer, as described below.

PL and GL molecular species were separated on a Shim-Pack diol column (4.6 mm × 50 mm, particle size 5 μm) (Shimadzu, Japan) using a Nexera-e chromatography system (Shimadzu, Japan). Solvent system A (2-propanol/hexane/H_2_O/HCOOH/(28%)NH_4_OH/Et_3_N, 28/72/1.5/0.1/0.05/0.02, v/v) and solvent system B (2-propanol/H_2_O/HCOOH/(28%)NH_4_OH/Et_3_N, 100/1.5/0.1/0.05/0.02, v/v) were used as eluents. The percentage of system B in the total solvent flow was programmed as follows: 0 to 20% (7 min), 20 to 100% (5 min), 100% (5 min), 100 to 0% (0.1 min), and 0% (10 min). Derivatized lipid extracts were separated on a Shim-Pack XR-SIL column (2.0 mm × 50 mm, particle size 2.2 μm) (Shimadzu, Japan) using a Nexera-e chromatography system (Shimadzu, Japan). Solvent system A (CHCl_3_/MeOH/(28%)NH_4_OH, 80/19.5/0.5, v/v) and solvent system B (CHCl_3_/MeOH/H_2_O/(28%)NH_4_OH, 60/34/5/0.5, v/v) were used as eluents. The percentage of system B in the total solvent flow was programmed as follows: 0 to 100% (20 min), 100% (30 min), 100 to 0% (10 min). The elution rate was 0.2 mL/min. Lipids were detected on a LCMS-IT-TOF high-resolution tandem mass spectrometer (Shimadzu, Japan). The analysis was performed in the electrospray ionization (ESI) mode with simultaneous registration of signals of positive and negative ions. Scanning was performed in a m/z range of 100–1200. The source voltage was –3.5 kV in the case of formation of negative ions and 4.5 kV in the case of formation of positive ions. The temperature of the ion source was 200 °C; the dry gas (N_2_) pressure, 200 kPa; the flow rate of nebulizing gas (N_2_), 1.5 L/min. Argon (0.003 Pa) was used in the collision chamber of the mass spectrometer. Molecular species of non-derivatized PL and GL were identified as described earlier^8^.

### Statistics and reproducibility

Values of lipid contents are presented as mean ± standard deviation for three biological samples (Supplementary Data 3). The raw data were used after being tested for normality of distribution (Shapiro–Wilk’s test). Significant differences between levels within the factors were determined by the analysis of variance (ANOVA) with the post hoc Tukey’s HSD test. A probability level of *p* < 0.05 was considered statistically significant. The preliminary data was arcsine-transformed^77^ prior to building of the heat maps. The heat maps were composed and all statistical analyses performed using the R statistical software (“rstatix” package for ANOVA and the post hoc Tukey’s HSD test, and “pheatmap” package for heat maps).

### Supplementary information

Description of Additional Supplementary Files

Supplementary Data 1

Supplementary Data 2

Supplementary Data 3

reporting-summary

## Data Availability

All relevant data generated and analyzed during this study can be found in Supplementary Data 1, 2 and 3.
