# Supplementary material for: Membrane vectorial lipidomic features of coral host cells’ plasma membrane and lipid profiles of their endosymbionts Cladocopium
Source: Commun Biol. 2024 Jul 18;7:878. doi: 10.1038/s42003-024-06578-8 (PMC11258240; doi:10.1038/s42003-024-06578-8)
Supplement: Supplementary file 1 — Description of Additional Supplementary Files [file 42003_2024_6578_MOESM1_ESM.pdf]

Description of Additional Supplementary Files

File name: Supplementary Data 1
Description: Supplementary information on molecular phylogenetic analysis

File name: Supplementary Data 2
Description: Estimates of evolutionary divergence between sequences of *Acropora* species

File name: Supplementary Data 3
Description: Profiles of the lipid molecular species of *Acropora aspera* and *Sinularia heterospiculata* host and symbionts
