# Supplementary material for: Membrane vectorial lipidomic features of coral host cells’ plasma membrane and lipid profiles of their endosymbionts Cladocopium
Source: Commun Biol. 2024 Jul 18;7:878. doi: 10.1038/s42003-024-06578-8 (PMC11258240; doi:10.1038/s42003-024-06578-8)
Supplement: Supplementary file 2 — Supplementary Data 1 [file 42003_2024_6578_MOESM2_ESM.pdf]

Membrane vectorial lipidomic features of plasma membrane of coral host cells and lipid profiles of their endosymbionts *Cladocopium*

Tatyana V. Sikorskaya[1*], Ekaterina V. Ermolenko[1], Taliya T. Ginanova[1], Andrey V. Boroda[1], Kseniya V. Efimova[1], Bogdanov Mikhail[2**]

[1]A.V. Zhirmunsky National Scientific Center of Marine Biology, Far Eastern Branch, Russian Academy of Sciences, Vladivostok, Russian Federation
[2]Department of Biochemistry and Molecular Biology, the University of Texas Health Science Center, McGovern Medical School, Houston, TX 77030, USA

*Lead contact, to whom correspondence should be addressed:
Tatyana V. Sikorskaya PhD
A.V. Zhirmunsky National Scientific Center of Marine Biology,
Far Eastern Branch of the Russian Academy of Sciences,
ul. Palchevskogo 17, 690041 Vladivostok, Russian Federation
Phone: +7(423)2310905, Fax: +7(423)2310900
E-mail: miss.tatyanna@yandex.ru
ORCID: https://orcid.org/0000-0003-1204-4433

**Corresponding author:
Mikhail Bogdanov, Ph.D.
Department of Biochemistry and Molecular Biology,
University of Texas Health Science Center, McGovern Medical School,
6431 Fannin St., Suite 6.204, Houston, TX 77030 USA

Tel: +1 (713)-500-6123

E-mail: mikhail.v.bogdanov@uth.tmc.edu

ORCID: https://orcid.org/0000-0002-7176-8127

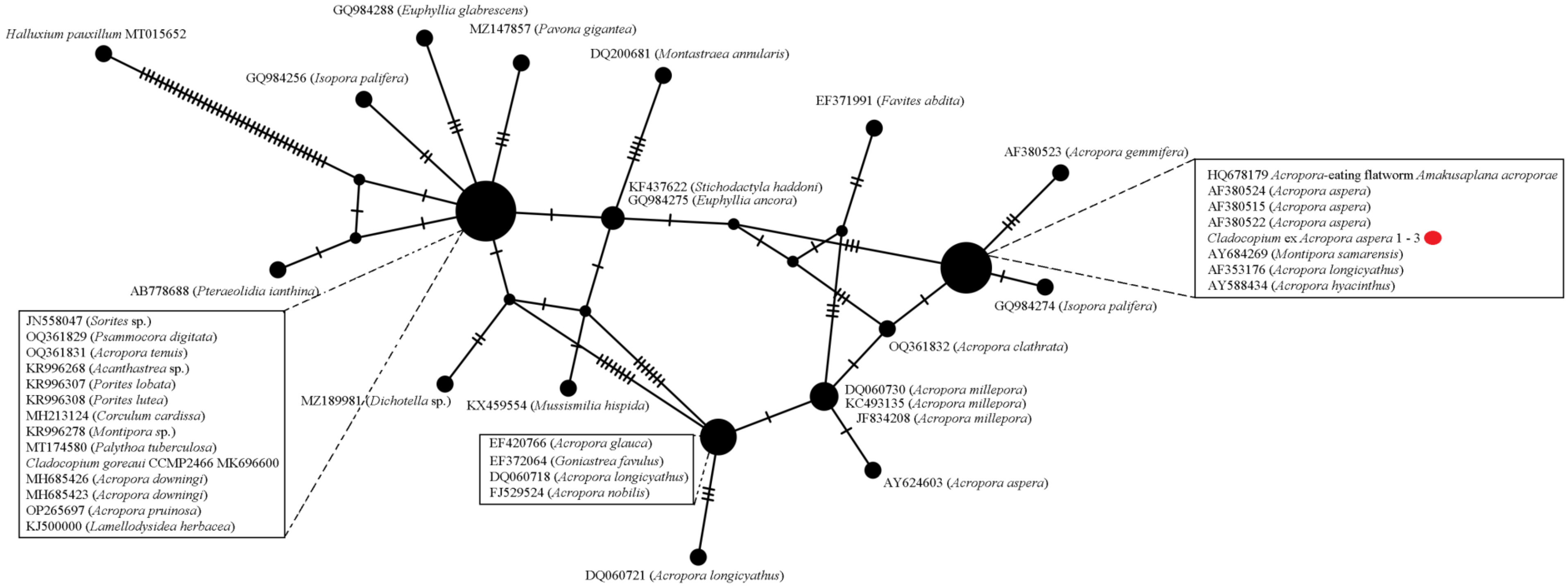

Fig. S1 Integer NJ Net network based on the LSU rRNA of *Cladocopium* sequences. Mutational steps are symbolized by dashes, and the diameter of the circles is proportional to the number of strains that belong to each ribotype. Sequences obtained in this study are highlighted in red dot.

Methods

Sequence alignment and molecular phylogenetic analyses

Details of the protocol of DNA extraction from fresh and ethanol-preserved coral colonies are provided in Sikorskaya et al. [1]. For species identification of *Acropora* sp. coral, direct sequencing of the highly polymorphic single-copy nuclear *Pax-C* 46/47 intron (*Pax-C*) was applied. The

nuclear *Pax-C* of *Acropora* was amplified with the PaxC_intron-FP1 (5′-TCCAGAGCAGTTAGAGATGCTGG-3′) and PaxC_intron-FR1 (5′-GGCGATTTGAGAACCAAACCTGTA-3′) primers [2].

The presence and specific species of Symbiodiniaceae were verified by PCR screening and sequencing of the obtained products according to the protocols and molecular-genetic markers published in Sikorskaya et al. [1,3]. The sequence data were deposited in GenBank under the following accession numbers: OR166010-OR166012, OR180049-OR180050.

Forward and reverse sequences were screened for quality, edited and assembled using the Geneious Prime software (v.2022.0.2, Biomatters Limited, Auckland, New Zealand). Alignment was performed using the MAFFT algorithm v7 [4] on https://mafft.cbrc.jp/alignment/server/.

A dataset on the *Pax-C* from 194 sequences of *Acropora*, including two sequences of *Isopora cuneata* as the outgroup, was preliminary constructed using the maximum likelihood (ML) method. The ML tree was generated using the IQ-TREE webserver [5], following the selection of substitution models using the Bayesian Information Criterion (BIC) in ModelFinder [6], which supported HKY+F+G4. Branch supports were assessed using the following options: 1000 replicates with SH-aLRT (approximate likelihood ratio test [aLRT] and Shimodaira–Hasegawa [SH]-aLRT) branch test [-alrt] [7], a bootstrap method based on analysis of 1000 UFBoot2 replicates [8] with a 0.99 minimum correlation as convergence criterion, and the Approximate Bayes test [-aBayes] [9]. To assess the number of putative species-level lineages within the *Acropora*, we implemented the automated species delineation method to estimate species boundaries from the *Pax-C* sequences: Assemble Species by Automatic Partitioning (ASAP) [10]. ASAP was run in webserver https://bioinfo.mnhn.fr/abi/public/asap/ using the Kimura (K80) distance model with TS/TV = 2.0. Inter-specific and intra-specific variabilities of the *Pax-C* gene of *Acropora* species were calculated in MEGA 11 [11] using the uncorrected *p*-distance model including transition and transversion substitutions and the homogeneous lineage pattern with the partial deletion option (less than 95% of site coverage was eliminated, i.e., fewer than 5% of alignment gaps, missing data, and ambiguous bases were allowed at any position).

We also used a dataset on the LSU rRNA from 51 sequences of *Cladocopium*, including the sequence of *Halluxium pauxillum* MT015652 as the outgroup. The Integer NJ Net algorithm of PopART v.1.7.2 software package [12] was used for the LSU rRNA network analysis of the *Cladocopium* sequences.

Results

Molecular phylogenetic analyses

Analysis of the nuclear *Pax-C* sequences indicated that the *Acropora* specimens shared 99–99.9% sequence similarity to the *Acropora aspera* sequences

from NCBI. For the ASAP analysis, ten resulting scenarios with asap scores/treshold dist (the lower the score the more reliable the results) were depicted in Fig. S2. The resulting ASAP analysis indicated that colonies of *Acropora* sp. from this analysis were most closely related to *A. aspera*, also supported by preliminary ML tree, and p-distances (Supplementary Table 1). The ASAP analysis revealed the "*Acropora aspera*" phylogroup as a single species. The level of differentiation of the LSU rDNA sequences of *A. aspera* and other closely related species was within the range of interspecific differences (Supplementary Table 1). The average intra-specific genetic divergence among *A. aspera* was 0.005 (0.5%). Our study shows the presence of one Symbiodiniaceae type, acroporide-specific *Cladocopium*, in all colonies. The Integer NJ Net network constructed based on the LSU rRNA dataset from *Cladocopium* sequences from this study and from GenBank is presented in Fig. S1. The LSU rRNA sequence shared 100% similarity to the published sequences of *Cladocopium* sp. from *Acropora*-eating flatworm *Amakusaplana acroporae* Rawlinson, Gillis, Billings & Borneman (HQ678179), *A. aspera* (AF380515, AF380522, AF380524), *A. longicyathus* Milne Edwards (AF353176), *A. hyacinthus* Dana (AY588434), and *Montipora samarensis* Nemenzo (AY684269).

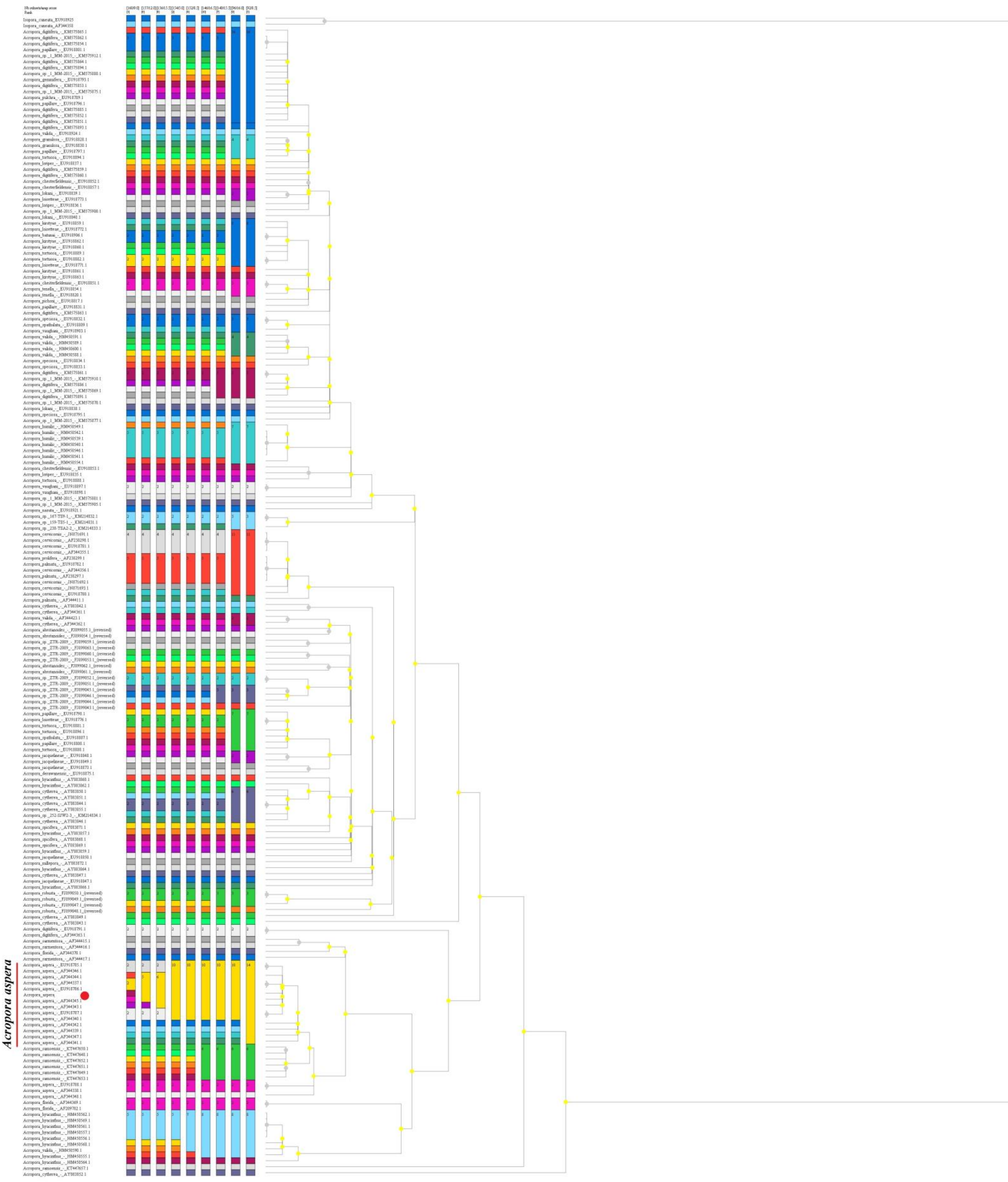

Fig. S2 Species delimitation based on the *Pax-C* sequences of *Acropora* generated by ASAP with ten resulting scenarios (colorful columns).

References

1. Sikorskaya, T.V., Efimova, K.V. and Imbs, A.B. (2021) Lipidomes of phylogenetically different symbiotic dinoflagellates of corals. Phytochemistry 181. 10.1016/j.phytochem.2020.112579
2. van Oppen, M.J., McDonald, B.J., Willis, B. and Miller, D.J. (2001) The evolutionary history of the coral genus Acropora (Scleractinia, Cnidaria) based on a mitochondrial and a nuclear marker: reticulation, incomplete lineage sorting, or morphological convergence? Mol Biol Evol 18, 1315-29. 10.1093/oxfordjournals.molbev.a003916
3. Sikorskaya, T.V., Ermolenko, E.V., Efimova, K.V. and Dang, L.T.P. (2022) Coral Holobionts Possess Distinct Lipid Profiles That May Be Shaped by Symbiodiniaceae Taxonomy. Mar Drugs 20. 10.3390/md20080485
4. Katoh, K., Rozewicki, J. and Yamada, K.D. (2019) MAFFT online service: multiple sequence alignment, interactive sequence choice and visualization. Brief Bioinform 20, 1160-1166. 10.1093/bib/bbx108
5. Nguyen, L.T., Schmidt, H.A., von Haeseler, A. and Minh, B.Q. (2015) IQ-TREE: A Fast and Effective Stochastic Algorithm for Estimating Maximum-Likelihood Phylogenies. Molecular Biology and Evolution 32, 268-274. 10.1093/molbev/msu300
6. Kalyaanamoorthy, S., Minh, B.Q., Wong, T.K.F., von Haeseler, A. and Jermiin, L.S. (2017) ModelFinder: fast model selection for accurate phylogenetic estimates. Nature Methods 14, 587-589. 10.1038/nmeth.4285
7. Guindon, S. et al. (2010) New Algorithms and Methods to Estimate Maximum-Likelihood Phylogenies: Assessing the Performance of PhyML 3.0. Systematic Biology 59, 307-321. 10.1093/sysbio/syq010
8. Hoang, D.T., Chernomor, O., von Haeseler, A., Minh, B.Q. and Vinh, L.S. (2018) UFBoot2: Improving the Ultrafast Bootstrap Approximation. Molecular Biology and Evolution 35, 518-522. 10.1093/molbev/msx281
9. Anisimova, M., Gil, M., Dufayard, J.F., Dessimoz, C. and Gascuel, O. (2011) Survey of Branch Support Methods Demonstrates Accuracy, Power, and Robustness of Fast Likelihood-based Approximation Schemes. Systematic Biology 60, 685-699. 10.1093/sysbio/syr041
10. Puillandre, N., Brouillet, S. and Achaz, G. (2021) ASAP: assemble species by automatic partitioning. Molecular Ecology Resources 21, 609-620. 10.1111/1755-0998.13281
11. Tamura, K., Stecher, G. and Kumar, S. (2021) MEGA11: Molecular Evolutionary Genetics Analysis Version 11. Mol Biol Evol 38, 3022-3027. 10.1093/molbev/msab120
12. Leigh, J.W. and Bryant, D. (2015) popart: full-feature software for haplotype network construction. Methods in Ecology and Evolution 6, 1110-1116. 10.1111/2041-210X.12410
